# Editorial Note: Assessment of time management practice and associated factors among primary hospitals employees in north Gondar, northwest Ethiopia

**DOI:** 10.1371/journal.pone.0336826

**Published:** 2025-11-18

**Authors:** The PLOS One Editors

This Editorial Note is issued to provide an update to the Expression of Concern previously issued on this article [1,2].

After the Expression of Concern [2] was issued, a discrepancy was noted between the study sites included in [1] and [3], despite reporting the same participant data. The authors have not responded to this concern or PLOS’ requests to provide the underlying data for either article.

Additionally, it was noted that the ethics approval for [1] is from an institution not listed in the affiliations list of [1]. The authors have not responded to PLOS’ inquiries regarding the ethics approval.
